# Bending space–time: a commentary on Dyson, Eddington and Davidson (1920) ‘A determination of the deflection of light by the Sun's gravitational field’

**DOI:** 10.1098/rsta.2014.0287

**Published:** 2015-04-13

**Authors:** Malcolm Longair

**Affiliations:** Cavendish Laboratory JJ Thomson Avenue, Cambridge CB3 0HE, UK

**Keywords:** general relativity, light bending by the Sun, eclipse expedition 1919, gravitational lensing, Eddington

## Abstract

The famous eclipse expedition of 1919 to Sobral, Brazil, and the island of Principe, in the Gulf of Guinea, led by Dyson, Eddington and Davidson was a turning point in the history of relativity, not only because of its importance as a test of Einstein's General Theory of Relativity, but also because of the intense public interest which was aroused by the success of the expedition. The dramatic sequence of events which occurred is reviewed, as well as the long-term impact of its success. The gravitational bending of electromagnetic waves by massive bodies is a subject of the greatest importance for contemporary and future astronomy, astrophysics and cosmology. Examples of the potential impact of this key tool of modern observational astronomy are presented. This commentary was written to celebrate the 350th anniversary of the journal *Philosophical Transactions of the Royal Society*.

## 1. Einstein and bent space–time

The famous expedition to measure the deflection of the positions of stars caused by the curvature of space–time in the gravitational field of the Sun had a profound impact upon the acceptance of the General Theory of Relativity and in arousing public interest in all things relativistic. Although it was not expressed in this way at the time, the concept that the fabric of the four-dimensional space–time we live in is determined by the distribution of mass–energy in the Universe lies at the heart of relativistic theories of gravity.

The story began in the late eighteenth century when non-Euclidean geometries started to be taken seriously by mathematicians who realized that Eudlid's fifth postulate—that parallel lines meet only at infinity—might not be essential for the construction of a self-consistent geometry [1,2].^1^ The fathers of non-Euclidean geometry were Nikolai Ivanovich Lobachevsky in Kazan in Russia and János Bolyai in Transylvania, then part of Hungary. In the 1820s, they independently solved the problem of the existence of non-Euclidean geometries and showed that Euclid's fifth postulate could not be deduced from the other postulates.

Non-Euclidean geometries were placed on a firm theoretical basis by the German mathematician Bernhard Riemann, and the English-speaking world was introduced to these ideas through the works of British mathematicians William Kingdon Clifford and Arthur Cayley. Until Einstein's discovery of the General Theory of Relativity, considerations of the geometry of space and the role of gravity in defining the structure of the Universe were separate questions. After 1915, they were inextricably linked.

The history of the discovery of General Relativity is admirably told by Abraham Pais in his scientific biography of Albert Einstein, *Subtle is the Lord: the Science and the Life of Albert Einstein* [3], in which many of the technical details of the papers published in the period 1907–1915 are discussed. Equally recommendable is the survey by John Stachel of the history of the discovery of both theories of relativity [4]. In seeking a fully self-consistent relativistic theory of gravity, Einstein was entering uncharted territory and for many years he ploughed a lone furrow, making the ultimate spectacular success of the theory in 1915 all the more remarkable.

It is simplest to quote Einstein's words from his Kyoto address [5] of December 1922:

In 1907, while I was writing a review of the consequences of Special Relativity,…I realised that all the natural phenomena could be discussed in terms of Special Relativity except for the law of gravitation. I felt a deep desire to understand the reason behind this…It was most unsatisfactory to me that, although the relation between inertia and energy is so beautifully derived [in Special Relativity], there is no relation between inertia and weight. I suspected that this relationship was inexplicable by means of Special Relativity.

He goes on:

I was sitting in a chair in the patent office in Bern when all of a sudden a thought occurred to me: ‘If a person falls freely he will not feel his own weight’. I was startled. This simple thought made a deep impression upon me. It impelled me towards a theory of gravitation.

In his comprehensive review of the Special Theory of Relativity published in 1907 [6], Einstein devoted the whole of the last section, Section V, to the *Principle of Relativity and Gravitation*. In the very first paragraph, he raised the question,

Is it conceivable that the principle of relativity also applies to systems that are accelerated relative to one another?

He had no doubt about the answer and stated the *Principle of Equivalence* explicitly for the first time:

…in the discussion that follows, we shall therefore assume the complete physical equivalence of a gravitational field and a corresponding acceleration of the reference system.

From this postulate, he derived the time-dilation formula in a gravitational field, d*t*=d*τ*(1+*ϕ*/*c*^2^), where *ϕ* is the gravitational potential, recalling that *ϕ* is always negative, *τ* is proper time and *t* is the time measured at zero potential. Then, applying Maxwell's equations to the propagation of light in a gravitational potential, he found that the equations are form-invariant, provided the speed of light varies in the gravitational potential as *c*(***r***)=*c*[1+*ϕ*(***r***)/*c*^2^], according to an observer at zero potential. Einstein realized that, as a result of Huyghens' principle, or equivalently Fermat's principle of least time, light rays are bent in a non-uniform gravitational field. He was disappointed to find that the effect was too small to be detected in any terrestrial experiment.

Einstein published nothing further on gravity and relativity until 1911, although he was undoubtedly wrestling with these problems through the intervening period. In his paper of that year [7], he reviewed his earlier ideas, noting that the gravitational dependence of the speed of light would result in the deflection of the light of background stars by the Sun. Applying Huyghens' principle to the propagation of light rays with a variable speed of light, he found the standard ‘Newtonian’ result that the angular deflection of light by a mass *M* would amount to Δ*θ*=2*GM*/*pc*^2^, where *p* is the collision parameter of the light ray. In the case of grazing incidence 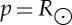 and the deflection amounted to 0.87 arcsec. Einstein urged astronomers to attempt to measure this deflection. Intriguingly, Einstein's prediction had been derived by Johann Georg von Soldner in 1801 on the basis of the Newtonian corpuscular theory of light [8].^2^

Following the famous first Solvay Conference of 1911 in Brussels, Belgium, Einstein returned to the problem of incorporating gravity into the Theory of Relativity and, from 1912 to 1915, his efforts were principally devoted to formulating the Relativistic Theory of Gravity. It was to prove to be a titanic struggle. In summary, his thinking was guided by four ideas:

— the influence of gravity on light,
— the principle of equivalence,
— Riemannian space–time, and
— the principle of covariance.

During 1912, he realized that he needed more general space–time transformations than those of Special Relativity. Two quotations illustrate the evolution of his thought.

The simple physical interpretation of the space-time coordinates will have to be forfeited, and it cannot yet be grasped what form the general space-time transformations could have [9].

If all accelerated systems are equivalent, then Euclidean geometry cannot hold in all of them. [5]

Towards the end of 1912, he realized that what was needed was non-Euclidean geometry. Einstein consulted his old school friend, the mathematician Marcel Grossmann, about the most general forms of transformation between frames of reference for metrics of the form d*s*^2^=g_*μν*_ d*x*^*μ*^ d*x*^*ν*^. Although outside Grossmann's field of expertise, he soon came back with the answer that the most general transformation formulae were the Riemannian geometries, but that they had the ‘bad feature’ that they are nonlinear. Einstein instantly recognized that, on the contrary, this was a great advantage since any satisfactory theory of Relativistic Gravity must be nonlinear.

The collaboration between Einstein and Grossmann was crucial in elucidating the features of Riemannian geometry, which were essential for the development of the General Theory of Relativity, Einstein fully acknowledging the central role which Grossmann had played. At the end of the introduction to his first monograph on General Relativity [10], Einstein wrote

Finally, grateful thoughts go at this place to my friend the mathematician Grossmann, who by his help not only saved me the study of the relevant mathematical literature but also supported me in the search for the field equations of gravitation.

The Einstein–Grossmann paper of 1913 [11] was the first exposition of the role of Riemannian geometry in the search for a Relativistic Theory of Gravity. The details of Einstein's struggles over the next 3 years are fully recounted by Pais [3]. It was a huge and exhausting intellectual endeavour which culminated in the presentation of the theory in its full glory to the Royal Prussian Academy of Sciences, Berlin, in November 1915. In that same month, Einstein discovered that he could account precisely for the perihelion shift of the planet Mercury.

In 1859, the French mathematician Urbain Le Verrier had discovered that, once account was taken of the influence of the planets, there remained an unexplained component of the advance of the perihelion of Mercury's elliptical orbit about the Sun, amounting to about 40 arcsec per century [12]. In a feat of technical virtuosity, Einstein showed in November 1915 that the advance of the perihelion of Mercury expected according to the General Theory of Relativity amounted to 43 arcsec per century, a value in excellent agreement with the present best estimates. This effect is a direct result of the distortion of space–time in the vicinity of the Sun. Einstein knew he must be right.

The theory also predicted the deflection of light by massive bodies because of the curvature of space–time in their vicinity. For the Sun, the predicted deflection of light rays from stars just grazing the limb of the Sun amounted to 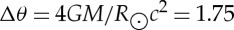 arcsec, a factor of 2 greater than that expected according to the Newtonian calculation. The reason for the factor of 2 difference is that both the space and time coordinates are bent in the vicinity of massive objects—it is four-dimensional space–time which is bent by the Sun. This prediction resulted in the famous eclipse expedition of 1919 led by Arthur Eddington and Andrew Crommelin, under the overall direction of the Astronomer Royal, Sir Frank Dyson.

## 2. Arthur Eddington

In 1902, Arthur Eddington won an entrance scholarship to read mathematics at Trinity College, University of Cambridge. He attended the lectures of the celebrated mathematicians E. T. Whittaker, A. N. Whitehead and E. W. Barnes, as well as being trained in the techniques for success in the Mathematical Tripos by the most distinguished mathematical coach of his time, R. A. Herman. In only his second year, he became Senior Wrangler,^3^ the first time a second-year student had attained that distinction.

In 1906, he took up the appointment of chief assistant at the Royal Greenwich Observatory, London, UK, where he obtained a thorough training in practical astronomy and began his theoretical studies in stellar dynamics. In 1912, he led an eclipse expedition to Brazil. In 1913, he returned to Cambridge as Plumian Professor of Astronomy and then Director of the Observatories—he was to remain in Cambridge for the rest of his career. Following Einstein's suggestion of 1911, a number of attempts had been made to measure the deflection of light by the Sun, but for a variety of reasons these had not been successful. Then, in a paper of March 1917 published in the *Monthly Notices of the Royal Astronomical Society* [13], Dyson pointed out that the total solar eclipse of May 1919 would be particularly favourable for testing the deflection of light by the Sun, which, according to Einstein's prediction, would be Δ*θ*=1.75 (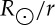) arcsec, where 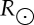 is the radius of the Sun and *r* is the projected distance of the star from the centre of the Sun. Not only was the totality of the eclipse unusually long, about 6 min, but also the Sun would then be observed against the background of the Hyades star cluster, providing many bright target stars suitable for the deflection experiments. Dyson urged preparations to be made for this outstanding and relatively rare opportunity.

Einstein's discovery of the General Theory of Relativity was communicated to the Berlin Academy of Sciences in 1915 [14]. Because of the First World War, direct communication with physicists in Germany was not possible, but the papers were forwarded to Eddington, who was then Secretary of the Royal Astronomical Society, by Willem de Sitter, a personal friend of Eddington's in neutral Holland. The theory is of considerable mathematical complexity, but, as Einstein stated in the last paragraph of his paper, ‘scarcely anyone who has fully understood this theory can escape from its magic’. Eddington was the ideal expositor of these ideas in English and within 2 years had written his *Report on the Relativity Theory of Gravitation* [15] for the Physical Society of London.^4^ Eddington's exposition of the theory was fully developed in his great book *The Mathematical Theory of Relativity* [16], which Einstein regarded as the best exposition of the theory in any language. Eddington had all the necessary experience of theory and observation for the eclipse expedition. The Joint Permanent Eclipse Committee obtained a grant of £100 for the instruments and £1000 for the expedition from the Government Grant Committee. A sub-committee consisting of Dyson, Eddington, Alfred Fowler, the distinguished Cambridge mathematical physicist, and Herbert Turner, Savilian Professor of Astronomy and Director of the Observatory at Oxford University, was appointed to make the arrangements for the 1919 expedition.

In 1917, conscription was enforced to support the War effort. As a Quaker, Eddington could not take human life and chose to become a conscientious objector. The intervention of Dyson persuaded the authorities to allow Eddington a 12 month deferment of his conscription to work on preparations for the 1919 eclipse expedition. As a result, Eddington was allowed to continue with his astronomical and astrophysical researches at a time when he was at the very peak of scientific creativity, as well as prepare for what turned out to be the post-War expedition.

## 3. The eclipse expeditions to Sobral and Principe

**Figure 1.**
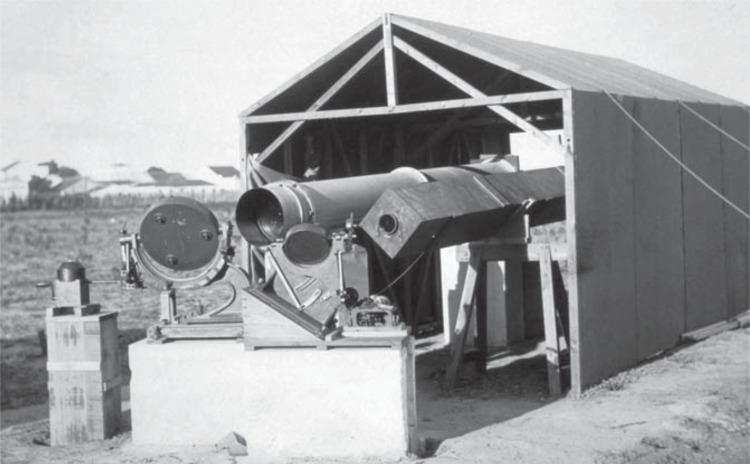
The 16-inch lens and large coelostat (on the left) and the smaller 4-inch telescope in a square box (on the right), with its much smaller coelostat on location at Sobral, Brazil. (Courtesy of the Science Museum, London.)

Reading the account of the 1919 eclipse expedition in *Philosophical Transactions* is like reading a *Boy's* *Own Paper* adventure story [17,18].^5^ The paper is full of colourful detail about the trials and tribulations of the expedition and it still makes delightful reading, as well as being a careful and judicious account of the results of the observations. The eclipse of 29 May 1919 passed over northern Brazil, across the Atlantic Ocean through the island of Principe and then across Africa. The Eclipse Committee decided on 10 November 1917 that there should be two expeditions, one to Sobral in northern Brazil and the other to the small island of Principe, lying off the west coast of Africa near Equatorial Guinea. Charles Davidson, assistant astronomer at the Royal Greenwich Observatory, and Father Aloysius Cortie, an expert on solar eclipses, were to take the 16-inch lens from the Oxford astrographic telescope to Sobral, while Eddington and Edwin Cottingham, a Northamptonshire clockmaker, took the 16-inch lens from the Greenwich astrographic telescope to Principe. Cottingham was responsible for overhauling all the clocks and the coelostats which were used to track the movement of the Sun during the eclipse—the 16-inch lenses were to remain in fixed positions inside long steel cylinders (figure 1). In February, Father Cortie suggested that they also take a small 4-inch telescope borrowed from the Royal Irish Academy with an 8-inch coelostat as a back-up to Sobral in case of problems. In the end, Father Cortie was unable to take part in the expedition and his place was taken by Andrew Crommelin, assistant astronomer at the Royal Greenwich Observatory.

The steamship *Anselm* took the adventurers first to the Island of Madeira, from where it continued on to South America with Davidson and Crommelin, while Eddington and Cottingham continued on the SS *Portugal* to Principe. Crommelin and Davidson set up their equipment in the covered grandstand at the racecourse of the Jockey Club at Sobral, whereas Eddington and Cottingham set up their equipment at Roça Sundy on a cocoa plantation at the northwest end of the island of Principe. It would spoil the reading of a splendid story to recount the various ups and downs of the expeditions, including strikes of workers and so on. Despite the experience of the team members and their careful preparations, the observations were not as successful as had been hoped. Nonetheless, important results were successfully obtained. In summary, the observations from the two stations were as follows:

**Principe (West Africa)**: as reported in the paper published in the *Philosophical Transactions of the Royal Society*

The days preceding the eclipse were very cloudy. On the morning of May 29 there was a very heavy thunderstorm from about 10 a.m. to 11.30 a.m.—a remarkable occurrence at that time of year. The sun then appeared for a few minutes, but the clouds gathered again. About half-an-hour before totality the crescent sun was glimpsed occasionally, and by 1.55 it could be seen continuously through drifting cloud. The calculated time of totality was from 2h. 13m. 5s. to 2h. 18m. 7s. G.M.T. Exposures were made according to the prepared programme, and 16 plates were obtained.

Most of the plates could not be used because the cloud cover was such that the stars could not be seen. During the last third of the eclipse conditions improved and a few stars suitable for the experiment could be used. Most weight was attached to two stars which were bright enough for astrometric determination of their positions to be feasible. The careful reduction of these data was carried out by Eddington in Cambridge,^6^ where the result 1.61±0.31 arcsec was obtained.

**Sobral (Brazil)**: the weather was better at Sobral, where both the 16-inch and 4-inch telescopes were used to take photographs throughout the eclipse (figure 1). The 16-inch telescope had to be stopped down to 8 inches because of serious astigmatism in the lens, and short exposures had to be used because of 30 s oscillations in the drive of the coelostat. But, as they wrote

…the results shown when the plates were developed were very disappointing. The images were diffused and apparently out of focus, although on the night of May 27 the focus was good. Worse still, this change was temporary, for without any changes in the adjustments, the instrument returned to focus when the comparison plates were taken in July.

These changes must be attributed to the effect of the Sun's heat on the mirror, but it is difficult to say whether this caused a real change of scale in the resulting photographs or merely blurred the images.

**Figure 2.**
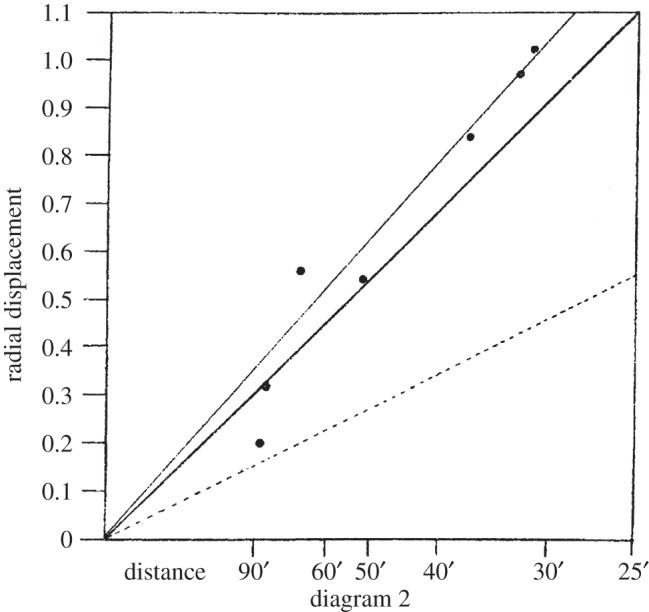
The radial deflections of the positions of seven stars observed by the 4-inch telescope at Sobral as a function of distance from the centre of the Sun. The scale on the abscissa is the inverse of the distance from the centre of the Sun. The dotted line shows the Newtonian prediction and the central heavy solid line shows the expectation of the General Theory of Relativity. The upper light solid line shows a best-fit to the deflection of the seven stars by the Sun. Image from [19] (Copyright The Royal Society).

Fortunately, the observations made with the back-up 4-inch telescope gave much better images and seven stars could be used to estimate the magnitude of the deflection. The reductions of the plates and the comparison plates were carried out at the Royal Greenwich Observatory by Davidson and Herbert Furner, a ‘computer’ at the Observatory, who found the result 1.98±0.12 arcsec. This result carried much more weight than that from the out-of-focus 16-inch lens, although the outward movement of the star positions caused by the presence of the Sun in the latter experiment was observed. Perhaps the most striking diagram illustrating the significance of the results is diagram 2 of the paper, which shows the deflections measured in the experiments as a function of distance from the centre of the Sun, compared with the expectations of the Newtonian (dotted line) and the Einstein (solid line) predictions (figure 2).

A source of some controversy was the fact that the paper describes attempts that were made to estimate the deflection using the blurred images from the Sobral 16-inch lens. The results depended upon whether or not there had been a change of scale of the plates because of heating of the coelostat mirror. If the change of scale were left as a free parameter in the analysis, a deflection of 0.93 arcsec was found; if it was assumed that there was no change of scale, the deflection was 1.52 arcsec. The authors of the paper considered that these data were too unreliable to be useful.

It is evident that all the other results were consistent with the prediction of Einstein's General Theory of Relativity of 1.75 arcsec and in poor agreement with the Newtonian expectation of 0.87 arcsec. The analysis was carried out meticulously by the team and they were duly cautious about their conclusions. They wrote:

Thus the results of the expeditions to Sobral and Principe can leave little doubt that a deflection of light takes place in the neighbourhood of the sun and that it is of the amount demanded by EINSTEIN's generalised theory of relativity, as attributable to the sun's gravitational field. But the observation is of such interest that it will probably be considered desirable to repeat it at future eclipses. The unusually favourable conditions of the 1919 eclipse will not recur, and it will be necessary to photograph fainter stars, and these will probably be at a greater distance from the sun.

## 4. The aftermath

### (a) The deflections

The results of the expedition were presented at a joint meeting of the Royal Society and the Royal Astronomical Society in London on 6 November 1919. The consistency with Einstein's prediction was indeed impressive. As the authors had stated, more data were needed and the next important opportunity was the 1922 eclipse observed at the Cordillo Downs sheep farm in South Australia, near the Queensland border. This time excellent data were obtained showing the displacements of many more stars, but the uncertainty remained stubbornly about 0.2–0.3 arcsec. The final result quoted in the paper by Dodwell & Davidson [20] was: 1.77±0.3 arcsec, again consistent with Einstein's prediction.

The issue of *Nature* published on 17 February 1921 was entirely devoted to papers concerning the status of the General Theory of Relativity, which had raised an enormous amount of interest in the scientific community as a result of the 1919 eclipse expedition. Dyson's paper on the expedition described the results of further analysis of the Sobral observations with the out-of-focus 16-inch lens [21]. He stated explicitly that:

If it is assumed that the scale has changed, then the Einstein deflection from the series of plates is 0.90 arcsec; if it is assumed that no real change of focus occurred, but merely a blurring of the images, the result is 1.56 arcsec; little weight is, however, attached to this series of photographs.

In 1979, at the request of the Director of the Royal Greenwich Observatory, Francis Graham Smith, and Andrew Murray, the senior astrometrist at the Observatory, and also to celebrate the centenary of Einstein's birth, the Sobral plates were reanalysed using Murray's astrometric data-reduction computer programs and new scans of the original plates and comparison plates made using their Zeiss Ascorecord plate-measuring machine [22]. These modern estimates confirmed the results of the 1919 analysis of the 4-inch plates, giving 1.90±0.11 arcsec. The blurred 16-inch astrometric data were also reanalysed with the result 1.55±0.34 arcsec, the larger errors reflecting the poorer quality of the plates.

### (b) Einstein and Eddington as public figures

One of the most remarkable aspects of the results of the expedition was the reaction of the general public to the discovery. The deflection of light by the Sun was trumpeted as a triumph for the General Theory of Relativity, which overturned many people's intuitive understanding of the nature of space and time. The day after the announcement of the results at the joint meeting of the Royal Society and the Royal Astronomical Society, the headline in *The Times* of London read ‘Revolution in Science. New Theory of Universe. Newtonian Ideas Overthrown’. J. J. Thomson, the President of the Royal Society and chairman of the joint meeting, was unambiguous about the importance of the observations. As reported in *The Times*,

Even the President of the Royal Society, in stating that they had just listened to ‘One of the most momentous, if not the most momentous, pronouncements of human thought’, had to confess that no one had yet succeeded in stating in clear language what the theory of Einstein really was.

This was indeed a challenge even for the scientifically literate since the theory is of considerable technical complexity. Eddington was undoubtedly the leading mathematical physicist in the UK who fully understood the significance and content of the General Theory and whose exposition was to become the standard presentation of the theory in English for many years [23]. Eddington became a much sought-after public lecturer, a role in which he excelled, conveying his personal vision of science and its accomplishments.

For Einstein, the effects were even more dramatic. As Pais notes in his biography of Einstein, before 1919, there was no mention of Einstein's name in *The New York Times*. After 1919, his name appears every year and became synonymous with the genius of theoretical physics whose thinking was on a quite different plane from other scientists, let alone the general public. This remarkable collaboration in science between the UK and Germany made some contribution to healing the wounds left by the First World War.

### (c) Radio astronomical tests of general relativity

Until the European Space Agency's (ESA) astrometry mission *Hipparcos* was launched in 1989, optical astrometry did not make much further progress in the measurement of the deflection of star positions by the Sun, largely because the ground-based star images are limited to about 1 arcsec in angular size because of the phenomenon of ‘seeing’, the broadening of their point images by refractive index fluctuations in the Earth's atmosphere.

**Figure 3.**
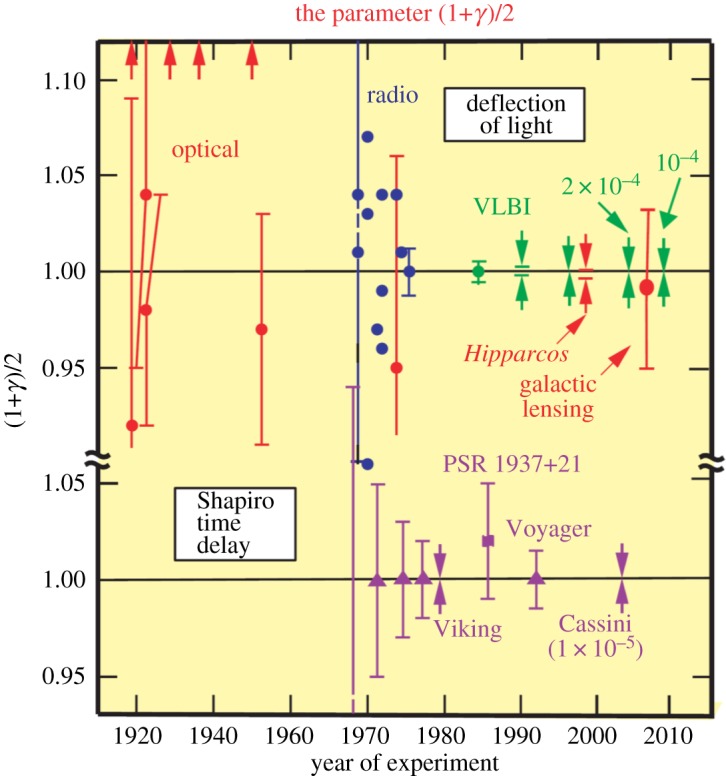
Measurement of the coefficient (1+*γ*)/2 from light deflection and time delay measurements [26]. In General Relativity, the value of *γ* is 1. The upper part of the diagram shows how the precision of the experiments has improved since the pioneering experiments of 1919. The lower part of the diagram shows the corresponding improvements in the limits to deviations from General Relativity from the Shapiro time delay experiments. (Courtesy of Prof. Clifford Will.)

In the meantime, the techniques of very-long baseline interferometry (VLBI) at radio wavelengths enabled angular differences at the milliarcsecond level to be measured. Of special importance were the radio quasars, many of which are very powerful point-like radio sources at cosmological distances and so provide an ideal extragalactic astrometric reference frame. A parametrized post-Newtonian formalism has been adopted in these and subsequent tests in which deviations of the parameters in the metric of space–time about the Sun are written as ‘corrections’ to General Relativity [24].^7^ For example, the parameter *γ* describes how much space curvature is produced by unit mass. In the case of the deflection of light by the Sun, the deflection is 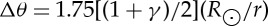 arcsec. In General Relativity, *γ*=1. Figure 3, taken from Will's review paper [24], shows how the precision of the deflection tests has improved up to 2014.

Among the first pairs of quasars to be used for this test were 3C273 and 3C279, both of which lie close to the plane of the ecliptic. In 1995, for example, the deviation of the predicted deflection from the Einstein prediction was found to be (1+*γ*)/2=0.9996±0.0017. Nowadays, with the use of VLBI for precise geodesy, accuracies at the submilliarc are routine. Even in the direction 90° from the Sun, the deflections amount to 4 milliarcsec, which is easily measurable. As a result, radio quasars all over the sky can be used to measure deflections of stars from the fundamental reference frame. In a 2004 analysis of almost 2 million observations of 541 radio sources made at 87 VLBI sites, the predicted deflections were found to agree with the Einstein prediction at the level (1+*γ*)/2=0.99992±0.00023. The accuracy of the best VLBI experiments is now better than 100 microarcsec.

In addition to these tests, radio astronomy contributed a fourth test—the delay of a radio signal from a transponder on a space vehicle or planet due to the fact that the radio signal passes through the gravitational potential well of the Sun. This *Shapiro effect* [25] depends in the same way as the deflection tests upon the parameter (1+*γ*)/2. The deviation of (1+*γ*)/2 from unity must be less than 0.0012% (figure 3).

**Figure 4.**
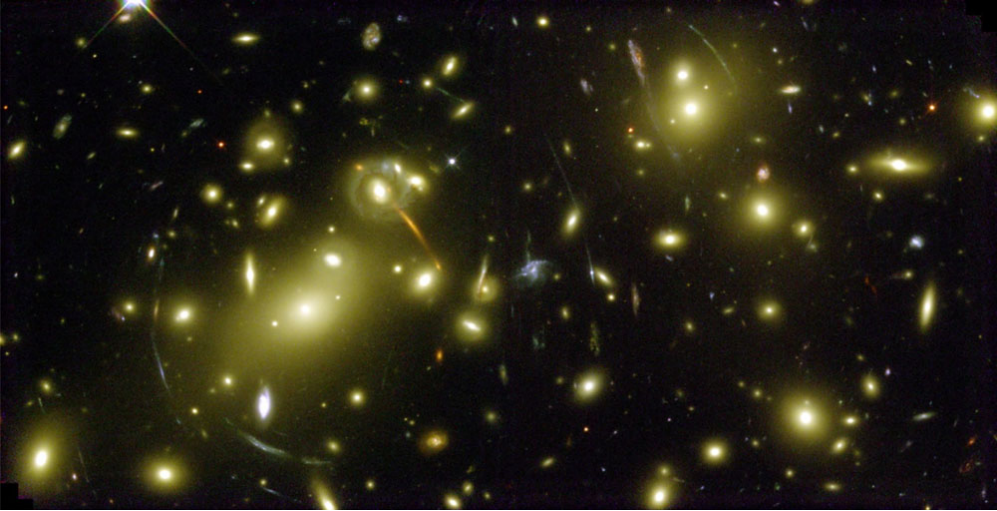
A Hubble Space Telescope image of the central region of the rich cluster of galaxies Abell 2218, showing the prominent arcs centred on the massive core of the cluster. The circular images are the gravitationally lensed images of a very distance background galaxy. The core of the cluster acts as a gravitational lens. (Courtesy of NASA, ESA and the Space Telescope Science Institute, Baltimore, MD, USA.)

There is now such confidence in the correctness of General Relativity that gravitational lensing has become a standard tool of the astronomer and cosmologist for studying such topics as the distribution of dark matter in the Universe and the detection of exoplanets about nearby stars. A spectacular example of gravitational lensing is provided by the Hubble Space Telescope images of rich clusters of galaxies, such as Abell 2218 (figure 4). While Einstein and Eddington had been somewhat cautious about the utility of gravitational lensing as an astronomical tool, in characteristic fashion, Fritz Zwicky, the great, if individual, Swiss astronomer, had no hesitation in realizing that the clusters of galaxies would act as huge gravitational lenses with perfectly measureable lensed arcs about the centre of the clusters [27]. It is no coincidence that Zwicky was also the first to measure the total mass of clusters of galaxies in 1933 and to show that there had to be much more hidden, or dark, matter present than would be inferred from the light of the cluster. In figure 4, incomplete arcs of ellipses are observed centred on the core of the cluster. For the case of stars, Einstein had predicted that if the star were perfectly aligned with a background star, the result would be what is often referred to as an *Einstein ring* [25]. These observations enable the total mass distribution in the cluster to be estimated and this is one of the most powerful means of estimating the amount of dark matter present.

There is no question, however, that optical astrometry and the deflection of light are very seriously back on the agenda. The turning point was the *Hipparcos* astrometric satellite of the European Space Agency [28],^8^ launched in 1989, which succeeded in measuring the positions of stars with milliarcsec accuracy, as well as pinning down the astrometric reference frame with this level of precision, tied to an extragalactic reference frame through quasar observations. The precision of the measurements was so high that the positions of stars all over the sky had to be corrected for the effects of the bending of light by the Sun and the planets. The resulting limit on (1+*γ*)/2 was within 0.3% of unity.

The European Space Agency's *Gaia* mission, launched in December 2013 and now in orbit at the Lagrangian L2 point, has the objective of measuring the positions of billions of stars with 20 microarcsec accuracy and so every observation will be subject to gravitational deflections. At the same time, these observations provide further ultra-high-precision tests of the General Theory of Relativity. The results of the 5 year *Gaia* mission should begin to appear about the time of the centenary of the 1919 eclipse expedition.

One of the most spectacular results of the application of gravitational lensing has been the derivation of the large-scale distribution of mass in the Universe from the ESA *Planck* satellite [29]. By measuring high-order correlations between the intensity distribution of the maps of the cosmological background radiation, the correlation function for dark matter has been measured and, combined with the other data from the mission, enables all the cosmological parameters to be determined from the one dataset.

Some measure of the importance of gravitational lensing for cosmology is that it will be central to the science of the ESA flagship mission *Euclid* and the NASA mission *WFIRST*, both of them designed to set constraints on the physics of the dark energy [30,31].^9^

## Footnotes

1 I have given a more detailed account of the origin of non-Euclidean geometries and the origins of General Relativity in my books [1,2].

2 In fact, Henry Cavendish appears to have come to essentially the same result as von Soldner in an unpublished manuscript of about 1784, inspired by John Michell's paper of the previous year on the escape of light from a massive body. Clifford Will [8] provides an intriguing comparison of how Cavendish and von Soldner may have each derived their result. Soldner's result can be found most easily from the deviation of particles under Rutherford scattering, for which cot Δ*θ*/2=(4*πε*_0_*m*/*zZe*^2^)*pv*_0_^2^, by replacing the electrostatic *zZe*^2^/4*πε*_0_ by the gravitational *GMm* and setting *v*_0_=*c* and . The limit Δ*θ*→0 is taken.

3 In the Cambridge Mathematical Tripos Examinations, the top student in mathematics is designated the ‘Senior Wrangler’. Normally the title would be awarded to the best-placed third-year student, but Eddington's remarkable ability gained him the top position after only 2 years of study.

4 Eddington's report was published in 1918 and a second edition in 1920 by the Physical Society of London, the latter including the results of the eclipse expedition of 1919.

5 The publications by Peter Coles and Daniel Kennefick give more details of this story at the non-technical level [17,18].

6 As noted in the paper published in the *Philosophical Transactions of the Royal Society*, ‘It had been intended to complete all the measurements of the photographs on the spot; but owing to a strike of the steamship company it was necessary to return by the first boat, if we were not to be marooned on the island for several months’.

7 The parametrized post-Newtonian formalism was described by Will & Nordtvedt [24].

8 For a comprehensive description of the astrometry and science achieved by the *Hipparcos* astrometry mission, see [28].

9 For more details about the role of gravitational lensing in astrophysics and cosmology, the following may be consulted: [30,31].

## Author profile

**Malcolm Longair** is Jacksonian Professor Emeritus of Natural Philosophy, University of Cambridge, and Director of Development at the Cavendish Laboratory, Cambridge. From 1980 to 1990, he was Astronomer Royal for Scotland and Director of the Royal Observatory, Edinburgh. From 1997 to 2005, he was Head of the Cavendish Laboratory. His research interests are in the areas of high-energy astrophysics and astrophysical cosmology, both very closely related to the subject of the present commentary. His recent interests have included the scientific history of physics, astronomy, astrophysics and cosmology. In 2013, he published a history of quantum mechanics and is currently writing a scientific history of the Cavendish Laboratory.
